# Existence of nontrival *n*-harmonic maps via min-max methods

**DOI:** 10.1007/s00526-026-03399-x

**Published:** 2026-07-03

**Authors:** Dorian Martino, Katarzyna Mazowiecka, Armin Schikorra

**Affiliations:** 1 Department of Mathematics, ETH Zurich, Rämistrasse 101, 8092 Zurich, Switzerland; 2 Institute of Mathematics, University of Warsaw, Banacha 2, 02-097 Warszawa, Poland; 3 Department of Mathematics, University of Pittsburgh, 301 Thackeray Hall, 15260 Pittsburgh, PA USA; 4 Department of Mathematics, Chulalongkorn University, Bangkok, Thailand

**Keywords:** 58E20, 35B65, 35J60, 35S05

## Abstract

For any n≥3 and any closed manifold ${\mathcal {N}}$ with $\pi _{n+k}({\mathcal {N}}) \ne \{0\}$ for some k≥0, we establish the existence of nontrivial *n*-harmonic maps from ${\mathbb {S}}^n$ into ${\mathcal {N}}$. When k≥1, these maps naturally appear as bubbling limits of *p*-harmonic maps with p>n, obtained by min-max constructions in the limit $p \rightarrow n^+$.

## Introduction

In this note, we study the existence of nontrivial *n*-harmonic maps from the sphere ${\mathbb {S}}^n$ into a smooth, compact manifold ${\mathcal {N}}\subset \mathbb {R}^N$ without boundary. These maps are critical points of the *n*-energy

$$ \mathcal {E}_n(u) :=\int _{{\mathbb {S}}^n} |\nabla u|^n,\quad u:{\mathbb {S}}^n \rightarrow {\mathcal {N}}, $$

i.e., distributional solutions to

1.1 $$\begin{aligned} -\textrm{div}(|\nabla u|^{n-2} \nabla u) = |\nabla u|^{n-2} A(u)[\nabla u,\nabla u]. \end{aligned}$$

Here *A* denotes the second fundamental form. Our main result is the following.

### Theorem 1.1

Let n≥3. If $\pi _{n+k}({\mathcal {N}}) \ne \{0\}$ for some k≥0, then there exists a nontrivial, $C^{1,\alpha }$-regular *n*-harmonic map from ${\mathbb {S}}^n$ into ${\mathcal {N}}$, where α is a small positive number.

Throughout the paper we assume n≥3. The two-dimensional case can be treated by the same arguments with minor modifications; since the corresponding results for harmonic maps are already well known (see [9] and references therein), we do not include them here.

The existence result in this theorem is in line with general expectations in the theory of harmonic maps. In the case k=0, that is, when $\pi _{n}({\mathcal {N}}) \ne \{0\}$, it follows from the classical Sacks–Uhlenbeck theory [19], since $\pi _{n}({\mathcal {N}})$ is generated by homotopy classes for which the *n*-energy admits a minimizer. The corresponding result for the *n*-harmonic maps was proved by [8, 17]. See also [13] for related minimization results in a fractional framework.

When $\pi _{n}({\mathcal {N}})= \{0\}$ but $\pi _{n+k}({\mathcal {N}}) \ni \alpha \ne 0$, one is naturally lead to seek critical points of the *n*-energy via a min-max construction of the form

$$ \inf _{\Phi \in \alpha }\ \max _{t\in [-1,1]^k}\ \mathcal {E}_n\big ( \Phi (\cdot ,t) \big ). $$

This is the equivalent approach to Birkhoff’s min-max construction of nontrivial closed geodesics on manifolds diffeomorphic to the sphere ${\mathbb {S}}^2$. A direct implementation of this strategy at the critical exponent *n* is, however, obstructed by the failure of the Palais–Smale condition in $W^{1,n}$.

In the recent work [3], this difficulty is overcome by developing a refined analytical framework, leading to a proof of the theorem Theorem 1.1 in dimension n=3, and in dimensions n≥4 in the case of manifolds which are spheres or Lie groups. The present work provides an alternative approach, which applies to all dimensions n≥3 and to arbitrary smooth closed target manifolds ${\mathcal {N}}$.

The starting point is classical. For p>n, we consider the perturbed energy

$$ \mathcal {E}_p(u) :=\int _{{\mathbb {S}}^n} |\nabla u|^p, \quad u:{\mathbb {S}}^n \rightarrow {\mathcal {N}}. $$

For p>n, the functional satisfies the Palais–Smale condition, allowing one to construct non-trivial critical points of the associated Euler–Lagrange equation

1.2 $$\begin{aligned} -\textrm{div}(|\nabla u|^{p-2} \nabla u) = |\nabla u|^{p-2} A(u)[\nabla u,\nabla u]. \end{aligned}$$

These are obtained through the min-max scheme

$$ \beta _p :=\min _{\Phi \in \alpha }\ \max _{ t\in [-1,1]^k}\ \mathcal {E}_p \big (\Phi (\cdot ,t) \big ). $$

Provided that $\liminf _{p \rightarrow n} \beta _p > 0$, Theorem 1.1 follows from a one-bubble compactness result: for any sequence $p \rightarrow n^+$, p>n and any corresponding sequence of *p*-harmonic maps $u_p \in W^{1,p}({\mathbb {S}}^n,{\mathcal {N}})$, one of the following holds, up to a subsequence. Either $u_p$ converges strongly in $W^{1,n+\sigma }$ for some σ>0, meaning that the limit is a nontrivial *n*-harmonic map; or there exists at least one nontrivial bubble, which, after rescaling, is a nontrivial *n*-harmonic map.

**Outline.** Since the case k=0 is known (see for instance [21, 22] or [8, Theorem 0]), we focus on the case k≥1. That is, throughout the remainder of this article we assume $\pi _{n+k}(\mathcal {N})\ne \{0\}$. In Section 2 we establish the validity of the min-max construction for *p*-harmonic maps and p>n. Section 3 explains the main analytical tool of the paper. There we prove an ε-regularity result for *p*-harmonic maps under a uniform *p*-energy bound. Instead of deriving higher *second-order* regularity, we obtain a fractional gain in the differentiability, showing that solutions belong locally to $W^{1+\delta , p}$ for some δ>0. This fractional improvement is sufficient to ensure the compactness needed in the bubbling analysis. The argument relies on the contemporary regularity theory for the *p*-Laplacian combined with the classical stability estimate in the spirit of Iwaniec–Sbordone [7], which provides a uniform control of the (n+σ)-energy for some σ>0.

Finally, in Section 4 we combine the previous results to prove the main theorem. Using the compactness provided by the fractional regularity improvement and a one-bubble extraction argument, we show the existence of a nontrivial *n*-harmonic map.

## Preliminaries: Existence of *p*-harmonic maps

Let $\alpha \in \pi _{n+k}(\mathcal {N})$ be nontrivial. We identify ${\mathbb {S}}^{n+k} \simeq {\mathbb {S}}^n \times [-1,1]^k$, with the usual identification on $\partial [-1,1]^k$, and consider for p>n

2.1 $$\begin{aligned} \inf _{\Phi \in \alpha }\ \max _{t \in [-1,1]^k}\ \mathcal {E}_p(\Phi (\cdot ,t))=\inf _{\Phi \in \alpha }\ \max _{t \in [-1,1]^k}\ \int _{{\mathbb {S}}^n} |\nabla \Phi (x,t)|^p \,\textrm{d}x{=}{:}\beta _p. \end{aligned}$$

We need the following.

### Lemma 2.1

Let $\beta _p$ be the value defined in (2.1). We have

$$\begin{aligned} \liminf _{p \rightarrow n^+} \beta _p > 0. \end{aligned}$$

### Proof

By Hölder inequality it suffices to show

$$ |{\mathbb {S}}^n|^{1-\frac{p}{n}} (\beta _p)^{\frac{n}{p}} \ge \inf _{\Phi \in \alpha } \max _{t \in [-1,1]^k} \int _{{\mathbb {S}}^n} |\nabla \Phi (x,t)|^n \,\textrm{d}x> 0. $$

Assume on the contrary that for a suitably small number ε>0 (chosen below) there exists a smooth $\Phi :{\mathbb {S}}^{n}\times [-1,1]^k \rightarrow {\mathcal {N}}$ for which we have

2.2 $$\begin{aligned} \max _{t \in [-1,1]^k} \int _{{\mathbb {S}}^n} |\nabla \Phi (x,t)|^n \,\textrm{d}x< \varepsilon . \end{aligned}$$

For each $t \in [-1,1]^k$, let $\Phi ^h(\cdot ,t)$ denote the harmonic extension of $\Phi (\cdot ,t):{\mathbb {S}}^n \rightarrow {\mathcal {N}}$ to a map $\Phi ^h(\cdot ,t):{B}^{n+1} \rightarrow \mathbb {R}^N$. If $P(x,\xi ) = \frac{1-|x|^2}{\omega _n |x-\xi |^{n+1}}$ is the Poisson kernel on the ball $B^{n+1}$, then we have

$$ \Phi ^h(x,t) = \int _{{\mathbb {S}}^n} P(x,\xi ) \Phi (\xi ,t)\,\textrm{d}\xi . $$

Since we have $\int _{{\mathbb {S}}^n}P(x,\xi )\,\textrm{d}\xi = 1$, the following holds for any $\zeta \in {\mathbb {S}}^n$

$$ \begin{aligned} \mathrm{dist\,}(\Phi ^h(x,t),{\mathcal {N}})&\le \left| \Phi ^h(x,t) - \Phi (\zeta ,t) \right| \le \int _{{\mathbb {S}}^n} P(x,\xi ) \left| \Phi (\xi ,t) - \Phi (\zeta ,t) \right| \,\textrm{d}\xi . \end{aligned} $$

Multiplying both sides by P(x,ζ) and integrating over ${\mathbb {S}}^n$ with respect to ζ we obtain

2.3 $$\begin{aligned} \begin{aligned} \mathrm{dist\,}(\Phi ^h(x,t),{\mathcal {N}})&\le \int _{{\mathbb {S}}^n}\int _{{\mathbb {S}}^n} P(x,\xi ) P(x,\zeta ) \left| \Phi (\xi ,t) - \Phi (\zeta ,t) \right| \,\textrm{d}\xi \,\textrm{d}\zeta . \end{aligned} \end{aligned}$$

If $|x|\le \frac{1}{2}$, then we have $|x-\xi |\ge \frac{1}{2}$ for any $\xi \in {\mathbb {S}}^n$ and thus, it holds

$$ \int _{{\mathbb {S}}^n}|P(x,\xi )|^{n'}\,\textrm{d}\xi \precsim \int _{{\mathbb {S}}^n}\left( \frac{1}{|x-\xi |^{n+1}} \right) ^{n'} \,\textrm{d}\xi \precsim 1. $$

Hence, for $|x|\le \frac{1}{2}$, we have, using Hölder and Poincaré inequality

$$ \begin{aligned}&\mathrm{dist\,}(\Phi ^h(x,t),{\mathcal {N}})\\&\le \Vert P(x,\cdot )\Vert _{L^{n'}({\mathbb {S}}^n)}^2 \left( \int _{{\mathbb {S}}^n}\int _{{\mathbb {S}}^n} \left| \Phi (\xi ,t) - \Phi (\zeta ,t) \right| ^n\,\textrm{d}\xi \,\textrm{d}\zeta \right) ^{\frac{1}{n}}\\&\precsim \left( \int _{{\mathbb {S}}^n}|\nabla \Phi (x,t)|^n \,\textrm{d}\xi \right) ^{\frac{1}{n}} \precsim \varepsilon ^{\frac{1}{n}}. \end{aligned} $$

If $|x|\ge \frac{1}{2}$, then we let r=1-|x| and $X=\frac{x}{|x|}$ and we write

$$ A(X,r,0) :=B(X,r), \quad A(X,r,k):=B(X,2^{k}r)\setminus B(X,2^{k-1}r) \text { for }k\ge 1. $$

Let us denote by $\theta = \mathrm{dist\,}_{{\mathbb {S}}^n}(\frac{x}{|x|},\xi )$. Then, we have

$$ |x-\xi |^2 = |x|^2+1-2|x|\frac{x}{|x|}\cdot \xi = |x|^2+1-2|x|\cos \theta = (1-|x|)^2+2|x|(1-\cos \theta ). $$

For every $\theta \in [0,1]$ we have the elementary inequality $\frac{2}{\pi ^2}\theta ^2\le 1-\cos \theta$, thus

$$ |x-\xi |^2\succsim (1-|x|)^2+|x|\theta ^2 \succsim (1-|x|)^2 + \theta ^2. $$

Hence, for $\xi \in A(X,r,k)$ we have $\theta \approx 2^k r$ and $|x-\xi |\succsim 2^k r$. Thus, from (2.3) we get for $|x|\ge \frac{1}{2}$

$$ \begin{aligned}&\mathrm{dist\,}(\Phi ^h(x,t),{\mathcal {N}}) \\&\precsim \sum _{k\in {\mathbb {N}}\cup \{0\}}\sum _{\ell \in {\mathbb {N}}\cup \{0\}}\int _{A(X,k,r)}\int _{A(X,\ell ,r)} \left| \Phi (\xi ,t) - \Phi (\zeta ,t) \right| \frac{r}{(2^k r)^{n+1}}\frac{r}{(2^\ell r)^{n+1}}\,\textrm{d}\xi \,\textrm{d}\zeta \\&\precsim \sum _{k\in {\mathbb {N}}\cup \{0\}}\sum _{\ell \in {\mathbb {N}}\cup \{0\}}2^{-k} 2^{-\ell }\bar{i}nt_{A(X,k,r)}\bar{i}nt_{A(X,\ell ,r)} \left| \Phi (\xi ,t) - \Phi (\zeta ,t) \right| \,\textrm{d}\xi \,\textrm{d}\zeta \\&\precsim \sum _{k\in {\mathbb {N}}\cup \{0\}}\sum _{\ell \in {\mathbb {N}}\cup \{0\}}2^{-k} 2^{-\ell } \left( \bar{i}nt_{A(X,k,r)}\bar{i}nt_{A(X,\ell ,r)} \left| \Phi (\xi ,t) - \Phi (\zeta ,t) \right| ^n\,\textrm{d}\xi \,\textrm{d}\zeta \right) ^\frac{1}{n}\\&\precsim \sum _{k\in {\mathbb {N}}\cup \{0\}}\sum _{\ell \in {\mathbb {N}}\cup \{0\}}2^{-k} 2^{-\ell } \Vert \nabla \Phi (\cdot ,t)\Vert _{L^n({\mathbb {S}}^n)} \precsim \varepsilon ^{\frac{1}{n}}. \end{aligned} $$

Thus, $$\left\| \mathrm{dist\,}(\Phi ^h,{\mathcal {N}}) \right\| _{L^{\infty }(B^{n+1}\times [-1,1]^k)} \precsim \varepsilon ^{1/n}$$.

Let $\pi _{{\mathcal {N}}}:B_{\delta }({\mathcal {N}}) \rightarrow {\mathcal {N}}$ denote the nearest point projection. We then define

$$ \Psi :=\pi _{{\mathcal {N}}} \circ \Phi ^h :{B}^{n+1} \times [-1,1]^k \rightarrow {\mathcal {N}}. $$

By construction, Ψ is continuous and satisfies $\Psi |_{{\mathbb {S}}^n\times [-1,1]^k} = \Phi .$ Hence, we have $\left[ \Psi |_{{\mathbb {S}}^n\times [-1,1]^k} \right] = [\Phi ]$ in $\pi _{k+n}({\mathcal {N}})$. On the other hand, the map $\Psi |_{{\mathbb {S}}^n\times [-1,1]^k}$ is contractible, therefore $\left[ \Psi |_{{\mathbb {S}}^n\times [-1,1]^k} \right] =[\Phi ]=0$. This is a contradiction with the assumption that $\alpha =[\Phi ]$ is nontrivial. □

Since p>n the functional $\mathcal {E}_p$ satisfies the Palais–Smale condition. In view of Lemma 2.1, we may apply the mountain-pass lemma^1^ (see, e.g., [20, Chapter II, Section 4, Theorem 4.2]) which yields the existence of a nontrivial critical point realizing the min-max value $\beta _p$. Since p>n, the resulting *p*-harmonic map $u_p$ is *Hölder continuous* by the Morrey–Sobolev embedding. Moreover, following the arguments of [5, Section 3] (see also [11, Theorem 1.3]) one concludes that $u \in C^{1,\alpha }$ for some α>0. We have thus established the following.

### Theorem 2.2

If $\pi _{n+k}({\mathcal {N}}) \ne \{0\}$ for some k≥1, then for any p>n there exists a *p*-harmonic map $u_p\in W^{1,p}\cap C^{1,\alpha }({\mathbb {S}}^n,{\mathcal {N}})$, i.e., a solution to (1.2), and we have

$$ \int _{{\mathbb {S}}^n} |\nabla u_p|^p =\beta _p=\min _{\Phi \in \alpha }\ \max _{t \in [-1,1]^k}\ \mathcal {E}_p\big (\Phi (\cdot ,t)\big ). $$

As a corollary, using a standard covering argument, see, e.g., [19, Proposition 4.3 and Theorem 4.4], we have the following disjunction of cases for the limiting behaviour of $u_p$ as $p\rightarrow n^+$.

### Corollary 2.3

Fix ε>0. If $\pi _{n+k}({\mathcal {N}}) \ne \{0\}$ for some k≥1, then there exists a sequence $p_j \xrightarrow {j \rightarrow \infty } n^+$ and a sequence of $p_j$-harmonic map $u\in W^{1,p_j}\cap C^{1,\alpha _j}({\mathbb {S}}^n,{\mathcal {N}})$, i.e., solutions to (1.2) with

$$ \liminf _{j \rightarrow \infty } \int _{{\mathbb {S}}^n} |\nabla u_{p_j}|^{p_j} > 0, $$

such that one of the following holds.

- (No bubble) For all $x \in {\mathbb {S}}^{n}$ there exists $\rho _x > 0$ such that
  $$ \limsup _{j \rightarrow \infty } \int _{B(x,\rho _x)} |\nabla u_{p_j}|^{n} < \varepsilon ^n. $$
- (At least one isolated bubble) There exists at least one $\bar{x} \in {\mathbb {S}}^n$ and a radius R>0 with the following property
  $$ \forall \rho > 0,\qquad \liminf _{j \rightarrow \infty } \int _{B(\bar{x},\rho )} |\nabla u_{p_j}|^{n} \ge \varepsilon ^n, $$
  and for any $x \in {\mathbb {S}}^n$, $0<\mathrm{dist\,}_{{\mathbb {S}}^n}(x,\bar{x})<R$ there exists some $\rho _x > 0$ such that
  $$ \limsup _{j \rightarrow \infty } \int _{B(x,\rho _x)} |\nabla u_{p_j}|^{n} \le \varepsilon ^n. $$

## An ε-regularity Theorem

The main technical ingredient of the paper is an ε-regularity theorem: it says that there is a threshold ε>0 such that for any (geodesic) ball B(x,ρ) where the *n*-energy drops below ε we have strong convergence (up to subsequence).

### Theorem 3.1

There exists an ε>0 such that for each $p_0>n$ there exist $p_1 > n$ and $t_0 > 0$ with the property that for all $p \in [n,p_1]$ and any Λ>0 the following holds.

Assume $u \in W^{1,p_0}(B(x_0,r),{\mathcal {N}})$ is a *p*-harmonic map which satisfies

$$ \Vert \nabla u\Vert _{L^n(B(x_0,r))} < \varepsilon \quad \text {and} \quad \Vert \nabla u\Vert _{L^p(B(x_0,r))} \le \Lambda . $$

Then it holds

$$ [\nabla u]_{W^{t_0,p}(B(x_0,r/2))} \le C(n,\Lambda ,r). $$

We begin with the following standard consequence of Iwaniec–Sbordone stability result [7, Theorem 4] for the *p*-Laplace equation.

### Lemma 3.2

(Iwaniec–Sbordone a priori estimate) Let n≥3, fix a compact manifold ${\mathcal {N}}\subset \mathbb {R}^N$ and let *A* be its second fundamental form. There exists ε>0, such that for any $p_0 > n$ there exists $p_1\in (n,p_0)$ and σ>0 so that the following holds whenever $p \in [n,p_1]$.

Assume that $u \in W^{1,p_0}(B(x_0,10r),{\mathcal {N}})$ satisfies

3.1 $$\begin{aligned} \textrm{div}(|\nabla u|^{p-2} \nabla u) = |\nabla u|^{p-2} A(u)[\nabla u,\nabla u] \quad \text {in } {B}(x_0,10r) , \end{aligned}$$

and

3.2 $$\begin{aligned} \Vert \nabla u\Vert _{L^n(B(x_0,10r))} < \varepsilon . \end{aligned}$$

Then it holds

$$ \Vert \nabla u\Vert _{L^{n+\sigma }(B(x_0,r))} \le C({\mathcal {N}},n)\left( 1+\Vert \nabla u\Vert _{L^{p}(B(x_0,10r))}^{p-1} \right) ^{\frac{n+1}{n-1}} . $$

### Proof

For simplicity of presentation, we assume that $B(x_0,10r)$ is a Euclidean ball *B*(0, 10)^2^.

First we localize. Let $\tilde{\eta } \in C_c^\infty (B(0,2))$ such that $\eta \equiv 1$ in *B*(0, 1) and set

$$ \tilde{u} :=\eta (u-(u)_{B(0,1)}). $$

We multiply (3.1) with $\eta ^{p-1}$ and obtain a system valid on $\mathbb {R}^n$

$$ \textrm{div}(|\nabla u|^{p-2} \nabla u)\eta ^{p-1} = |\nabla u|^{p-2} A(u)[\nabla u,\nabla u]\eta ^{p-1} \quad \text {in } \mathbb {R}^n . $$

Thus, it holds

3.3 $$\begin{aligned} \begin{aligned} \textrm{div}(\eta ^{p-1} |\nabla u|^{p-2} \nabla u)&= |\nabla u|^{p-2} A(u)[\nabla u,\nabla u]\eta ^{p-1} + (p-1) \eta ^{p-2} \nabla \eta |\nabla u|^{p-2} \nabla u\\&= |\nabla \tilde{u}|^{p-2} A(u)[\nabla \tilde{u},\nabla u] + g,\\ \end{aligned} \end{aligned}$$

where the map *g* verifies the following estimate

3.4 $$\begin{aligned} \Vert g\Vert _{L^{p'}(\mathbb {R}^n)} \precsim \Vert \nabla u\Vert _{L^p(B(0,2))}^{p-1}, \end{aligned}$$

where the constant depends on $\Vert u\Vert _{L^\infty }$, that is on $\mathcal {N}$.

We define the following quantity

$$ F:=|\nabla \tilde{u}|^{p-2} \nabla \tilde{u}- \eta ^{p-1} |\nabla u|^{p-2} \nabla u . $$

Since p>2, the map *F* satisfies the following estimate

3.5 $$\begin{aligned} \Vert F\Vert _{L^{\frac{p}{p-2}}(\mathbb {R}^n)} \precsim \Vert \nabla u\Vert _{L^p(B(0,2))}^{p-2}, \end{aligned}$$

where the constant depends on $\Vert u\Vert _{L^\infty }$, that is on $\mathcal {N}$.

Plugging *F* into (3.3), we arrive at

3.6 $$\begin{aligned} \begin{aligned} \textrm{div}(|\nabla \tilde{u}|^{p-2} \nabla \tilde{u})&= |\nabla \tilde{u}|^{p-2} A(u)[\nabla \tilde{u},\nabla u] + g + \textrm{div}F. \end{aligned} \end{aligned}$$

Since by assumption $\tilde{u} \in W^{1,p_0}(B(0,3))$, then for some $0<\sigma \le p_0-p$ with σ<1, we can find (see [6, Theorem 8.1] and [7, Theorem 4]) via Hodge decomposition $\varphi \in W^{1,\frac{p+\sigma }{1+\sigma }}_0(B(0,3))$ and $B \in L^{\frac{p+\sigma }{1+\sigma }}(B(0,3))$ such that

$$ |\nabla \tilde{u}|^{\sigma } \nabla \tilde{u} = \nabla \varphi + B \quad \text {in { B}(0,\,3)}, $$

with the estimates

3.7 $$\begin{aligned} \Vert \nabla \varphi \Vert _{L^{\frac{p+\sigma }{1+\sigma }}(B(0,3))} \precsim \Vert \nabla \tilde{u}\Vert _{L^{p+\sigma }(B(0,3))}^{1+\sigma } \end{aligned}$$

and

3.8 $$\begin{aligned} \Vert B\Vert _{L^{\frac{p+\sigma }{1+\sigma }}(B(0,3))} \precsim \sigma \Vert \nabla \tilde{u}\Vert _{L^{p+\sigma }(B(0,3))}^{1+\sigma }. \end{aligned}$$

The constants in inequalities (3.7) and (3.8) are independent of *p* and σ, provided p∈[n,n+1] and $\sigma \in [0,1]$.

Since $\mathrm{supp\,}\tilde{u} \subset B(0,3)$, we have

$$ \begin{aligned}&\Vert \nabla \tilde{u} \Vert _{L^{p+\sigma }(\mathbb {R}^n)}^{p+\sigma }\\&=\int _{B(0,3)} |\nabla \tilde{u}|^{p-2} \nabla \tilde{u} \cdot \nabla \varphi + \int _{B(0,3)} |\nabla \tilde{u}|^{p-2} \nabla \tilde{u} \cdot B\\&\le \left| \int _{B(0,3)} |\nabla \tilde{u}|^{p-2} \nabla \tilde{u} \cdot \nabla \varphi \right| + \Vert \nabla \tilde{u} \Vert _{L^{p+\sigma }(B(0,3))}^{p-1} \Vert B\Vert _{L^{\frac{p+\sigma }{1+\sigma }}(B(0,3))}\\&\le C\left| \int _{B(0,3)} |\nabla \tilde{u}|^{p-2} \nabla \tilde{u} \cdot \nabla \varphi \right| + C\sigma \Vert \nabla \tilde{u} \Vert _{L^{p+\sigma }(\mathbb {R}^n)}^{p+\sigma }. \end{aligned} $$

The constant *C* is independent of σ. Thus for $\sigma = (2C)^{-1}$, we can absorb the second term on the right-hand side, and arrive at

3.9 $$\begin{aligned} \Vert \nabla \tilde{u} \Vert _{L^{p+\sigma }(\mathbb {R}^n)}^{p+\sigma } \precsim \left| \int _{B(0,3)} |\nabla \tilde{u}|^{p-2} \nabla \tilde{u} \cdot \nabla \varphi \right| , \end{aligned}$$

with constants depending on *n*, but uniform in p∈[n,n+1]. For the fixed $\sigma = (2C)^{-1}$ that is not going to change anymore, let $p_1 \in (n,n+1)$ be so that

$$ \frac{p_1+\sigma }{1+\sigma } < n. $$

This is possible because n≥2. Then we have

3.10 $$\begin{aligned} \sup _{p \in [n,p_1]} \frac{p+\sigma }{1+\sigma } < n . \end{aligned}$$

Using (3.9) (where constants are independent of $p \in [n,p_1]$) together with (3.6), we obtain

3.11 $$\begin{aligned} \begin{aligned} \Vert \nabla \tilde{u} \Vert _{L^{p+\sigma }(\mathbb {R}^n)}^{p+\sigma } \precsim&\left| \int _{B(0,3)} |\nabla \tilde{u}|^{p-2} \nabla \tilde{u} \cdot \nabla \varphi \right| \\ \precsim&\int _{B(0,3)} |\nabla u| |\nabla \tilde{u}|^{p-1} \left| \varphi \right| +\int _{B(0,3)} \left| g \right| \left| \varphi \right| +\int _{B(0,3)} \left| F \right| \left| \nabla \varphi \right| . \end{aligned} \end{aligned}$$

We apply Hölder inequality to the first term with exponents

$$ \frac{1}{n} + \frac{p-1}{p+\sigma } + \frac{1}{q} = 1, \quad \text {where} \quad \frac{1}{q} = 1-\frac{p-1}{p+\sigma } - \frac{1}{n} = \frac{1+\sigma }{p+\sigma } - \frac{1}{n}\le 1. $$

Hence, we obtain that $q = \left( \frac{p+\sigma }{1+\sigma }\right) ^*$. By the Sobolev–Poincaré inequality (which we can apply with constant independent of *p* because (3.10) holds) and by (3.7) we get

3.12 $$\begin{aligned} \begin{aligned} \int _{B(0,3)} |\nabla u| |\nabla \tilde{u}|^{p-1} \left| \varphi \right|&\le \Vert \nabla u \Vert _{L^n(B(0,3))}\, \Vert \nabla \tilde{u} \Vert _{L^{p+\sigma }(\mathbb {R}^n)}^{p-1} \, \Vert \varphi \Vert _{L^{\left( \frac{p+\sigma }{1+\sigma }\right) ^* }(B(0,3))} \\&\precsim \Vert \nabla u \Vert _{L^n(B(0,3))}\, \Vert \nabla \tilde{u} \Vert _{L^{p+\sigma }(\mathbb {R}^n)}^{p-1} \, \Vert \nabla \varphi \Vert _{L^{ \frac{p+\sigma }{1+\sigma } }(B(0,3))} \\&\precsim \Vert \nabla u \Vert _{L^n(B(0,3))}\, \Vert \nabla \tilde{u} \Vert _{L^{p+\sigma }(\mathbb {R}^n)}^{p+\sigma }. \end{aligned} \end{aligned}$$

We also have $\left( \frac{p+\sigma }{1+\sigma }\right) ^*>p$. Indeed, the inequality is equivalent to $\frac{p+\sigma }{1+\sigma }>\frac{np}{n+p}$, which in turn is equivalent to

$$ p^2+\sigma (n-(n-1)p)>0. $$

Since $p\ge n\ge 2$, we have n-(n-1)p≤0, and using 0<σ<1 we obtain

$$ p^2+\sigma (n-(n-1)p) \ge p^2+(n-(n-1)p) = p(p-n)+n+p >0. $$

This proves the claimed inequality $\left( \frac{p+\sigma }{1+\sigma }\right) ^*>p$.

Hence, we obtain using (3.7) and then (3.4)

3.13 $$\begin{aligned} \begin{aligned} \int _{B(0,3)} \left| g \right| \left| \varphi \right|&\le \Vert g\Vert _{L^{p'}(B(0,3))}\, \Vert \varphi \Vert _{L^p(B(0,3))} \\&\precsim \Vert g\Vert _{L^{p'}(B(0,3))}\, \Vert \nabla \varphi \Vert _{L^{\frac{p+\sigma }{1+\sigma }}(B(0,3))}\\&\precsim \Vert \nabla u\Vert _{L^p(B(0,2))}^{p-1}\Vert \nabla \tilde{u}\Vert _{L^{p+\sigma }(B(0,3))}^{1+\sigma }. \end{aligned} \end{aligned}$$

To estimate the last term of (3.11), we note that since σ<1 we have $\frac{p}{2} \le \frac{p+\sigma }{1+\sigma }$. Hence, from Hölder inequality, (3.7), and (3.5) we obtain

3.14 $$\begin{aligned} \begin{aligned} \int _{B(0,3)} \left| F \right| \left| \nabla \varphi \right|&\le \Vert F\Vert _{L^{\frac{p}{p-2}}(B(0,3))}\Vert \nabla \varphi \Vert _{L^{\frac{p}{2}}(B(0,3))} \precsim \Vert \nabla u\Vert _{L^p(B(0,2))}^{p-2} \Vert \nabla \tilde{u}\Vert _{L^{p+\sigma }(B(0,3))}^{1+\sigma }. \end{aligned} \end{aligned}$$

Combining (3.11) with (3.12), (3.13), and (3.14) we obtain

$$ \begin{aligned}&\Vert \nabla \tilde{u} \Vert _{L^{p+\sigma }(\mathbb {R}^n)}^{p+\sigma } \precsim \ \Vert \nabla u\Vert _{L^n(B(0,3))} \Vert \nabla \tilde{u}\Vert _{L^{p+\sigma }(\mathbb {R}^n)}^{p+\sigma } \\&\quad + \left( \Vert \nabla u\Vert _{L^{p}(B(0,3))}^{p-1} +\Vert \nabla u\Vert _{L^{p}(B(0,3))}^{p-2} \right) \Vert \nabla \tilde{u}\Vert _{L^{p+\sigma }(\mathbb {R}^n)}^{1+\sigma }. \end{aligned} $$

With the smallness assumption (3.2), and Young’s inequality for exponents $\frac{1+\sigma }{p+\sigma } + \frac{p-1}{p+\sigma } =1$ with δ>0, we find for all $p \in [n,p_1]$

$$ \begin{aligned} \Vert \nabla \tilde{u} \Vert _{L^{p+\sigma }(\mathbb {R}^n)}^{p+\sigma } \le&\ C(n,\mathcal {N}) \left( \varepsilon \Vert \nabla \tilde{u}\Vert _{L^{p+\sigma }(\mathbb {R}^n)}^{p+\sigma } + \delta \Vert \nabla \tilde{u}\Vert _{L^{p+\sigma }(\mathbb {R}^n)}^{p+\sigma } \right) \\&+ C(\delta ,n,\mathcal {N}) \left( \Vert \nabla u\Vert _{L^{p}(B(0,3))}^{p-1} +\Vert \nabla u\Vert _{L^{p}(B(0,3))}^{p-2} \right) ^{\frac{p+\sigma }{p-1}}. \end{aligned} $$

Now, since $\frac{p+\sigma }{p-1} \le \frac{n+1}{n-1}$ for $p\in [n,p_1]$, provided that δ and ε are small enough, we obtain

$$ \Vert \nabla \tilde{u} \Vert _{L^{p+\sigma }(\mathbb {R}^n)}^{p+\sigma } \precsim _{\delta ,n} \left( 1+\Vert \nabla u\Vert _{L^{p}(B(0,3))}^{p-1} \right) ^{\frac{n+1}{n-1}}. $$

In particular, since $\eta \equiv 1$ in *B*(0, 1),

$$ \Vert \nabla u \Vert _{L^{n+\sigma }(B(0,1))}^{n+\sigma } \precsim _{\delta ,n} \left( 1+\Vert \nabla u\Vert _{L^{p}(B(0,3))}^{p-1} \right) ^{\frac{n+1}{n-1}}. $$

□

We combine Lemma 3.2 with the following result obtained in [1, Theorem 4.1]. See [1, Remark 4.2] for arbitrary dimension and systems.

### Theorem 3.3

Let n≥2. There exists $s_0 = s_0(n)> 0$ such that the following holds. Let p∈[n,n+1], $f \in L_{loc}^{1}(B(0,2))$ and $u \in W^{1,p}(B(0,2),\mathbb {R}^N)$ be a solution to

$$ \textrm{div}(|\nabla u|^{p-2} \nabla u) = f \quad \text {in } B(0,2) \subset \mathbb {R}^n . $$

Then for any $s \in (0,s_0]$ and $c_0>1$ such that $\frac{np'}{n+(1-s)p'} \ge c_0$, there exists a constant $C = C(n,s,c_0)>0$ such that the following holds

3.15 $$\begin{aligned} \left[ |\nabla u|^{p-2} \nabla u \right] _{W^{s,p'}(B(0,1))} \le C\,\Vert f\Vert _{L^{\frac{np'}{n+(1-s)p'}}(B(0,2))} + C\, \Vert \nabla u\Vert _{L^{p}(B(0,2))}^{p-1}. \end{aligned}$$

In the above result, the dependence of the constants $s_0$ and *C* can be chosen continuously in p∈[n,n+1] by following the proof of [1, Theorem 4.1], taking into account [1, Remark 4.2]. Additionally, inequality (3.15) corresponds to inequality [1, (4.2)] after applying the Sobolev embedding. Now Theorem 3.1 follows from Theorem 3.3 combined with Lemma 3.2.

### Proof of Theorem 3.1

Again, for simplicity of presentation, we may assume that $B(x_0,r)$ is a Euclidean ball *B*(0, 100).

Again, as in Section 2, since the exponent $p_0$ from the statement of the theorem satisfies $p_0>n$, Morrey’s embedding together with [5, Section 3] (or [11, Theorem 1.3]) yields $u\in C^{1,\alpha }$ for some α>0, and in particular $\nabla u\in L^\infty$. Consequently, the parameter $p_0$ appearing in Lemma 3.2 can be chosen arbitrarily above *n*; for instance, taking $p_0=n+1$ in Lemma 3.2, we obtain the existence of $\sigma \in (0,1)$ and $p_1\in (n,n+1)$ such that, if $p\in [n,p_1]$ we have

$$ \Vert \nabla u\Vert _{L^{n+\sigma }(B(0,20))} \precsim _\Lambda 1. $$

Possibly shrinking $p_1$, we may further assume that $\frac{n+\sigma }{p}>\frac{2n+\sigma }{2n}>1$ for all $p\in [n,p_1]$. With this choice, *u* satisfies

$$ \textrm{div}\bigl (|\nabla u|^{p-2}\nabla u\bigr )=f, $$

where

3.16 $$\begin{aligned} \Vert f\Vert _{L^{\frac{n+\sigma }{p}}(B(0,20))} \precsim \Vert \nabla u\Vert _{L^{n+\sigma }(B(0,20))}^{p} \precsim _\Lambda 1. \end{aligned}$$

Let $s_0>0$ be given by Theorem 3.3. Shrinking $s_0$ if necessary, we may assume that

$$ 0<s\le s_0 \quad \Longrightarrow \quad s<\frac{n\sigma }{2n+\sigma }. $$

Fix such an *s*. Then, for all $p\in [n,p_1]$,

$$ \frac{n}{n-s}\le \frac{2n+\sigma }{2n} \qquad \text {and} \qquad \frac{np'}{n+(1-s)p'}\le \frac{2n+\sigma }{2n} < \frac{n+\sigma }{p}. $$

Moreover, by possibly shrinking $p_1$ once more so that $p_1<\frac{n}{1-s}$, we ensure that

$$ \frac{np'}{n+(1-s)p'}>1 \qquad \text {for all } p\in [n,p_1]. $$

Hence, we first apply (3.15), then Hölder inequality, and finally (3.16), which yields

$$ \bigl [\,|\nabla u|^{p-2}\nabla u\,\bigr ]_{W^{s,p'}(B(0,15))} \precsim _\Lambda \Vert f\Vert _{L^{\frac{n+\sigma }{p}}(B(0,30))}+1 \precsim _\Lambda 1. $$

Moreover, the following inequality holds for p≥2 (see for instance [10, Lemma 2.3.7])

$$ \left| |\nabla u|^{p-2}(x) \nabla u(x)-|\nabla u|^{p-2}(y) \nabla u(y) \right| \succsim _{p} \left| \nabla u(x)-\nabla u(y) \right| ^{p-1}. $$

We find that

$$ \int _{B(0,15)}\int _{B(0,15)} \frac{\left| \nabla u(x)- \nabla u(y) \right| ^{p}}{|x-y|^{n+\frac{s}{2(p-1)} p}}\, dx\, dy \precsim _{\Lambda }1. $$

Setting $t_0 = \frac{s}{2(p_1-1)} \le \frac{s}{2(p-1)}$ we conclude. □

## Conclusion: Proof of Theorem 1.1

### Proof of Theorem 1.1

Let $p_0=n+1$ and let us take ε from Theorem 3.1. Recall that we assume that k≥1 (because k=0 is known already). That is, we can apply Corollary 2.3 to find a sequence $p_j \xrightarrow {j \rightarrow \infty } n^+$ and a sequence of nontrivial $p_j$-harmonic maps $u_{p_j} \in C^{1,\alpha _j}({\mathbb {S}}^n,{\mathcal {N}})$. There are two possible cases.

Case 1: There are no bubbles, i.e., for all $x \in {\mathbb {S}}^n$ there exists $\rho _x > 0$ such that

$$ \limsup _{j \rightarrow \infty } \int _{B(x,\rho _x)} |\nabla u_{p_j}|^{n} < \varepsilon ^n. $$

In this case we cover the compact set ${\mathbb {S}}^n$ by finitely many balls $B(x,\rho _x/2)$, apply Theorem 3.1 on each $B(x,\rho _x/2)$, and from the embedding $W^{t_0,p_j}_{loc} \hookrightarrow W^{\frac{t_0}{2},n}_{loc}$ obtain^3^

$$ \sup _j [\nabla u_{p_j}]_{ W^{\frac{t_0}{2},n}({\mathbb {S}}^n)} < \infty . $$

Thus, up to a subsequence,

$$ u_{p_j} \rightarrow u \quad \text{ strongly } \text{ in } W^{1,n+\sigma } \text { for some } \sigma > 0 \text {.} $$

By Vitali’s convergence theorem we then have

$$ \int _{{\mathbb {S}}^n} |\nabla u|^n = \lim _{j \rightarrow \infty } \int _{{\mathbb {S}}^n} |\nabla u_{p_j}|^{p_j} >0. $$

By the strong convergence, we can also pass to the limit in the equation (1.2) and conclude that *u* is an *n*-harmonic map. The map *u* cannot be trivial since $\Vert \nabla u\Vert _{L^n} > 0$. Since $u \in W^{1,n+\gamma }$ we know that *u* is Hölder continuous. Thus, as in [5, Section 3], $u \in C^{1,\alpha }$ is a nontrivial *n*-harmonic map.

Case 2: There is an isolated bubble, i.e., there exists $\bar{x} \in {\mathbb {S}}^n$ such that:

4.1 $$\begin{aligned} \forall \varrho >0, \qquad \liminf _{j\rightarrow \infty } \int _{B(\bar{x},\varrho )} |\nabla u_{p_j}|^n \ge \varepsilon ^n, \end{aligned}$$

4.2 $$\begin{aligned} \limsup _{j\rightarrow \infty } \int _{{\mathbb {S}}^n} |\nabla u_{p_j}|^n \le M < \infty , \end{aligned}$$

and for some R>0 and any $x \in \overline{B(\bar{x},R)} \setminus \{\bar{x}\}$ there exists $\rho _x > 0$ such that

4.3 $$\begin{aligned} \limsup _{j\rightarrow \infty } \int _{B(x,\rho _x)} |\nabla u_{p_j}|^n \le \varepsilon ^n . \end{aligned}$$

Up to subsequence we then may conclude from Theorem 3.1

4.4 $$\begin{aligned}  &   u_{p_j} \xrightarrow {j \rightarrow \infty } u \quad \text {weakly in } W^{1,n}({\mathbb {S}}^n) , \end{aligned}$$

4.5 $$\begin{aligned}  &   u_{p_j} \xrightarrow {j \rightarrow \infty } u \quad \text {strongly in } W^{1,n}_{loc}\!\left( B(\bar{x},R)\setminus \{\bar{x}\}\right) . \end{aligned}$$

We argue as in [2, Proof of Lemma 1] and [12, Section 5.2] (see also [15]). Up to composition with a smooth diffeomorphism — for instance the exponential map centered at $\bar{x}$ followed by a dilation — we may assume that $u_{p_j}$ and *u* are defined on the Euclidean unit ball *B*(0, 1), with $\bar{x}=0$ and R=1. In these coordinates the maps are $p_j$-harmonic with respect to the pull-back metric *g* induced by $\mathbb {S}^n$. Since we are analyzing concentration at $\bar{x}=0$, the coefficients of *g* converge in $C^\infty _{\textrm{loc}}$ to the Euclidean metric $\delta _{ij}$ near 0. Throughout the remainder of this section we keep the metric *g* implicit and do not record it explicitly in the $L^p$ norms.

For $\bar{R} :=\frac{1}{4} >0$, we define

$$ Q_j(\rho ) :=\sup _{x\in B(0,\bar{R})} \int _{B (x,\rho )} |\nabla u_{p_j}|^{n}. $$

We note that $Q_j$ is continuous, $Q_j(0)=0$, non-decreasing in ρ, and by (4.1) for large *j*

$$ \begin{aligned} Q_j(2\bar{R})&= \sup _{x\in B (0,\bar{R})}\int _{B (x,2\bar{R})} |\nabla u_{p_j}|^{n} \ge \int _{B (0,\bar{R})}|\nabla u_{p_j}|^{n}\ge \varepsilon ^{n}. \end{aligned} $$

Thus, there exists $\rho _j>0$ and $x_j \in \overline{B (0,\bar{R})}$ such that

$$ Q_j(\rho _j) = \int _{B (x_j,\rho _j)} |\nabla u_{p_j}|^n = \frac{\varepsilon ^n}{2}. $$

We show that $x_j\rightarrow 0$ and $\rho _j\rightarrow 0$. Up to a subsequence, assume $x_j\rightarrow \bar{y}\in \overline{B(\bar{x},\bar{R})}$ and $\rho _j\rightarrow \bar{\rho }\ge 0$. If $\bar{\rho }\ne 0$, then for *j* large,

$$ \frac{\varepsilon ^n}{2}=Q_j(\rho _j)\ge \int _{B(0,\rho _j)}|\nabla u_{p_j}|^n \ge \int _{B(0,\bar{\rho }/2)}|\nabla u_{p_j}|^n\ge \varepsilon ^n, $$

a contradiction; hence $$\rho _j\rightarrow 0$$.

If $\bar{y}\ne \bar{x}$, let *u* be the limit from (4.4). Choose $\rho \in (0,\rho _y)$ (where $\rho _y$ is defined in (4.3)) so that

$$ \int _{B(\bar{y},\rho )}|\nabla u|^n<\frac{\varepsilon ^n}{4}, \qquad \bar{x}\notin B(\bar{y},2\rho ), $$

and *j* large enough so that $2(|\bar{y}-x_j|+\rho _j)\le \rho$. Then

$$ \begin{aligned} \frac{\varepsilon ^{n}}{2}&= \int _{B (x_j,\rho _j)} |\nabla u_{p_j}|^{n} \le \int _{B \big (\bar{y}, 2(|\bar{y}-x_j|+\rho _j) \big )} |\nabla u_{p_j}|^{n} \le \int _{B (\bar{y},\rho )} |\nabla u_{p_j}|^{n}. \end{aligned} $$

By (4.5),

$$ \frac{\varepsilon ^n}{2} \le \lim _{j\rightarrow \infty }\int _{B(\bar{y},\rho )}|\nabla u_{p_j}|^n =\int _{B(\bar{y},\rho )}|\nabla u|^n <\frac{\varepsilon ^n}{4}, $$

a contradiction; thus $x_j\rightarrow \bar{x}$, hence $x_j\rightarrow 0$ and $\rho _j\rightarrow 0$.

Define now

$$ \Omega _j :=\frac{ B (0,\bar{R}) -x_j }{\rho _j} \subset \mathbb {R}^n , \qquad v_j(x) = u_{p_j}\left( x_j + \rho _j\, x \right) \text { for } x\in \Omega _j. $$

Then we have $\Omega _j \rightarrow \mathbb {R}^n$ in the sense that for any R>0 there exists *j* large enough so that $B (0,R) \subset \Omega _j$. Also $v_j:(\Omega _j,g(x_j + \rho _j\cdot ))\rightarrow {\mathcal {N}}$ is a $p_j$-harmonic map (because it satisfies the equation (1.2), which is scaling invariant).

For *j* large enough $B (0,1)\subset \Omega _j$ and we have

4.6 $$\begin{aligned} \int _{B (0,1)} |\nabla v_j|^{n} = \int _{B (x_j,\rho _j)} |\nabla u_{p_j}|^{n} = Q_j(\rho _j) = \frac{\varepsilon ^{n}}{2}. \end{aligned}$$

For any $x\in \mathbb {R}^n$ we have for *j* large enough $B (x,1)\subset \Omega _j$ and since $\rho _j\rightarrow 0$ and $x_j \rightarrow 0$ there exists $y\in B (0,\bar{R})$ such that for sufficiently large *j* we have $B (x_j+\rho _j x,\rho _j) \subset B (y,\rho _j)$. Hence, it holds

$$ \int _{B (x,1)} |\nabla v_j|^{n} = \int _{B (x_j+\rho _j x,\rho _j)} |\nabla u_{p_j}|^{n} \le \sup _{y\in B (0,\bar{R})}\int _{B (0,\bar{\rho }_j)} |\nabla u_{p_j}|^{n} = Q_j(\rho _j) = \frac{\varepsilon ^{n}}{2}. $$

Thus, for any $x\in \mathbb {R}^n$ and sufficiently large *j* we have

$$ \int _{B (x,1)} |\nabla v_j|^{n} \le \frac{\varepsilon ^{n}}{2}. $$

Since this holds for any $x \in \mathbb {R}^n$, up to passing to a subsequence, we can assume

$$ v_j \xrightarrow {j \rightarrow \infty } \omega \quad \text {weakly in } W^{1,n}_{loc}(\mathbb {R}^n) . $$

Moreover, from Theorem 3.1, combined with Sobolev embedding, we obtain that for every $x\in \mathbb {R}^n$

$$ v_j \rightarrow \omega \text { strongly in } W^{1,n+\sigma }\left( B (x,1/2 )\right) . $$

Since $v_j$ are $p_j$ harmonic maps, with the strong $W^{1, n+\sigma }-$ convergence we may pass to the limit in the equation of $v_j$ and obtain that the limiting map $\omega \in W^{1,n}_{loc}(\mathbb {R}^n,{\mathcal {N}})$ also satisfies an *n*-harmonic map equation in $\mathbb {R}^n$.

Moreover, ω is non-trivial. Indeed, by the change of the variables (and the scaling invariance of the *n*-energy)

$$ \int _{B (0,1)}|\nabla \omega |^n = \lim _{j\rightarrow \infty } \int _{B (0,1)} |\nabla v_j|^n = \lim _{j\rightarrow \infty }\int _{B (x_j,\rho _j)} |\nabla u_{p_j}|^n = \lim _{j\rightarrow \infty } Q_j(\rho _j) = \frac{\varepsilon ^n}{2} \ne 0. $$

In addition, we find that ω has finite energy. Indeed, for any R>0 we have

$$ \int _{B (0,R)} |\nabla \omega |^n = \lim _{j\rightarrow \infty } \int _{B (0,R)} |\nabla v_j|^n = \lim _{j\rightarrow \infty } \int _{B (x_j,\rho _j R)} |\nabla u_{p_j}|^n \le C(n,M), $$

where the last inequality is the uniform bound on the *n*-energy of $u_{p_j}$ from (4.2). Thus, it holds $\nabla \omega \in L^n(\mathbb {R}^n)$. We also observe that as $W^{1,n+\sigma }$-limit ω is Hölder continuous, and since it solves the *n*-harmonic map equation it is actually $C^{1,\alpha }(\mathbb {R}^n,{\mathcal {N}})$ for some α>0.

In order to find a nontrivial harmonic map $\tilde{\omega }:{\mathbb {S}}^n \rightarrow {\mathcal {N}}$ we argue as follows. Let $\tau _N:{\mathbb {S}}^n\setminus \{N\}\rightarrow \mathbb {R}^n$ be the stereographic projection from the north pole. By conformal invariance of the *n*-harmonic map equation in the domain, the map $\tilde{\omega } :=\omega \circ \tau _N :{\mathbb {S}}^n\setminus \{N\}\rightarrow {\mathcal {N}}$ is *n*-harmonic on ${\mathbb {S}}^n\setminus \{N\}$ and satisfies $\tilde{\omega }\in W^{1,n}({\mathbb {S}}^n\setminus \{N\},{\mathcal {N}})\cap C^{1,\alpha }({\mathbb {S}}^n\setminus \{N\},{\mathcal {N}})$.

To remove the singularity at *N*, consider the stereographic projection $\tau _S:{\mathbb {S}}^n\setminus \{S\}\rightarrow \mathbb {R}^n$ from the south pole and define $\omega _2 :=\tilde{\omega }\circ \tau _S^{-1}:\mathbb {R}^n\setminus \{0\}\rightarrow {\mathcal {N}}$. Then $\omega _2$ is *n*-harmonic on $\mathbb {R}^n\setminus \{0\}$ and $\omega _2=\omega \circ (\tau _N\circ \tau _S^{-1})=\omega \circ \kappa$, where $\kappa (x)=\frac{x}{|x|^2}$. Since κ is conformal and *n* is the critical exponent, the *n*-energy is invariant under κ, and we have $\Vert \nabla \omega _2\Vert _{L^n(B(0,1))} = \Vert \nabla \omega \Vert _{L^n(\mathbb {R}^n\setminus B(0,1))}$.

$$ \int _{B(0,1)} |\nabla \omega _2|^n = \int _{\mathbb {R}^n\setminus B(0,1)} |\nabla \omega |^n <\infty , $$

so $\omega _2$ has finite *n*-energy in a neighborhood of the isolated singularity at 0. By the removable singularity theorem [16, Theorem 5.1] (see also [4]), $\omega _2$ extends to a map in $C^{1,\alpha }(\mathbb {R}^n,{\mathcal {N}})$ (hence also in $W^{1,n}_{\textrm{loc}}(\mathbb {R}^n,{\mathcal {N}})$). Reverting the stereographic projection, we conclude that $\tilde{\omega }$ extends to an *n*-harmonic map $\tilde{\omega }\in W^{1,n}({\mathbb {S}}^n,{\mathcal {N}})\cap C^{1,\alpha }({\mathbb {S}}^n,{\mathcal {N}})$. Moreover, $\tilde{\omega }$ is non-constant since ω is non-constant. □

## Data Availability

Data sharing not applicable to this article as no datasets were generated or analysed during the current study.

## Footnotes

1 The space $W^{1,p}({\mathbb {S}}^n,{\mathcal {N}})$ is a Banach manifold of class $C^{\infty }$ when p>n, see for instance the proof of Proposition II.1 in [18]. Moreover, the functional $\mathcal {E}_p$ is $C^1$ as long as p>1.

2 In the case of a geodesic ball in ${\mathbb {S}}^n$, one works in normal coordinates; the *n*-Laplacian then has smooth coefficients, and all arguments and estimates carry over unchanged.

3 For s∈(0,1) in general there is *no* embedding $W^{s,p}_{\textrm{loc}}\hookrightarrow W^{s,q}_{\textrm{loc}}$ when p>q; see [14].

## Declarations

### Notation.

Throughout the paper, *B*(*x*, *r*) denotes the (open) ball of radius r>0 centred at *x*. When the ambient space is $\mathbb {R}^n$, this is the Euclidean ball; when the ambient space is ${\mathbb {S}}^n$, it is the geodesic ball. The meaning will always be clear from the context.
